# Quality of life of adolescents living with sickle cell anaemia in Ondo State, Nigeria

**DOI:** 10.11604/pamj.2020.35.124.19082

**Published:** 2020-04-15

**Authors:** Funmilola Adenike Faremi, Oyeninhun Abimbola Olawatosin

**Affiliations:** 1 Department of Nursing Science, Obafemi Awolowo University, Ile-Ife, Nigeria; 2 Department of Nursing, University of Ibadan, Ibadan, Nigeria

**Keywords:** Knowledge, quality of life, adolescent, sickle cell disease

## Abstract

**Introduction:**

This study assessed the level of knowledge and quality of life of adolescents living with sickle cell disease (SCD) and their quality of life.

**Methods:**

This study utilized descriptive research design. The study was conducted in two secondary level hospitals with functional SCD clinic in Ondo State. One hundred and four (104) adolescents participated in the study. Their consents were sought and gained.

**Results:**

Results showed that more males (63.5%) participated in the study. The mean age of the participants was 15.26±3.09 years. More than half (62.5%) of the participants that participated in the study had SCD crisis within six months. The mean SCD knowledge of participants in the study was 22.12±2.76, while 15.4% of the participants had good knowledge of SCD and Only 13(12.5%) have high quality of life. Also, the mean score of the participants on the quality of life scale was 39.50±6.47.

**Conclusion:**

Knowledge of adolescents with SCD that participated in the study was relatively low and their quality of life moderate. Effort should therefore, geared towards improving their knowledge about SCD and providing supportive care that will improve their quality of life.

## Introduction

Sickle Cell Anaemia (SCA) is an inherited blood disorder that affects the haemoglobin within the red blood cells [1]. SCA was first described by Herrick in 1910 and it is the commonest genetic disease in most countries in sub-Saharan Africa. Globally, about 100 million people, predominantly blacks are affected by the disease [2]. Over 300,000 babies are born with SCA worldwide mostly in low and middle income countries, although higher-income areas like Europe and America are not excluded; with majority of births in Africa thus making it a global health problem [3]. Generally, the prevalence of carriers ranges between 10% to 40% across equatorial Africa and 1% to 2% in Northern Africa, with less than 1% in Southern Africa [4]. In East African countries, such as Tanzania and Uganda, a wide variation of about 45% was reported; while in West African countries such as Ghana and Nigeria, the frequency of carriers' ranges from 15% to 30% [5]. In Nigeria, 24% of the populations are carriers of the mutant gene and the prevalence of sickle cell anaemia is about 20 per 1000 live births, which implies that in Nigeria alone, about 150,000 children are born annually with SCA [5]. World Health Organization report indicates that the sickle-cell trait is now known to be widespread, reaching its highest prevalence in parts of Africa as well as among people with origins in equatorial Africa, the Mediterranean basin and Saudi Arabia [1]. Sickle cell anaemia is a condition that has profound consequences on the quality of life (QoL) of sufferer and their family member [5]. Quality of life implies how an individual perceive his/her position in life in terms of culture and value system, and how it relates to set goals, expectations, standards and concerns. SCA affects persons across ages; it is a lifelong disorder that requires attention at each developmental stage. Adolescents with SCA often experience a wide range of complications including severe pain, chronic anaemia and jaundice, susceptibility to infection, pulmonary complications and acute chest syndrome, stroke risk, short stature and delayed puberty [6, 7]. These complications tend to compromise adolescents' health status, quality of life, and emerging independence [8]. This may interfere with the important developmental process of adolescent transition to adulthood and adult SCA medical care [7]. A good knowledge of the disease and the disease process on the part of the adolescent is expected to reduce the impact of this disease on their quality of life and health status. This study is therefore, designed to evaluate the knowledge of SCA among adolescent living with SCA in Ondo State and their quality of life.

## Methods

This study utilized descriptive research design; quantitative method of data collection was used to obtain data from the respondents. The study was conducted among adolescents living with SCA in Ondo State, Nigeria. Two health facilities with functional SCA clinic were purposively selected for the study. Ethical approval was sought and gained from the Research and Ethical Committee of Ondo State Hospitals Management Board. The two hospitals had a total of 150 adolescents who were duly registered as members of the SCD clinics. However, only 104 adolescents were accessible to the researchers. Consent was taken from the 104 accessible populations. For adolescents who were less than 18 years of age, parent/guardian assent were also obtained. Adapted SCD Transition Knowledge Questionnaire was used to assess teen knowledge of SCA [9]. It has a calculated alpha coefficient of 0.79 while adapted Sickle Cell Quality of Life scale (SC-QoL) [10] assessed adolescents' quality of life. The reliability of research instrument was assessed with Cronbach Alpha, with the following values respectively SC-QoL: 0.80 and SCD knowledge: 0.83. The questionnaires were pre-coded for ease of analysis. The 15 questions that were used to assess SCD knowledge were scored by allocating one (1) mark to every correct answers and zero (0) to incorrect answers giving a total obtainable score of 15 (100%). Scores were categorized into poor, good and very good knowledge. Scores below the 25^th^ percentile were classified as poor knowledge, and scores between the 25^th^ to 50^th^ percentile were classified as good knowledge while scores greater than 50^th^ percentile were classified as very good knowledge. Quality of life of adolescents living with SCA were scored using 15 item questions adapted from SCD-QoL Scale which was used to determine adolescents' quality of life. The 15 items scores ranges from (1 = Always to 4 = Never). Scores were transformed to a 0 to 100 scale which is common in most quality of life measures. Also, scores were categorized into poor Qol, moderate Qol and good Qol. Scores below the 25^th^ percentile were classified as poor quality of life and scores between the 25^th^ to 50^th^ percentiles were classified as moderate quality of life while score greater than 50^th^ percentile were classified as good quality of life. Frequencies and percentages of the demographic variables were presented in tables and charts. Mean and standard deviation was used for continuous variables and correlation coefficient was used to test relationship between gender and quality of life.

## Results

Findings revealed that more males (63.5%) participated in the study, also majority of the respondents were Christians (81.0%), and Yoruba (91.3%). Respondents' ages were reported as follows 10-14 years (35.6%), 15-16 years (22.1%), while 17-19 years were 42.3%, this classification was done according to World Health Organization classification of adolescence. The mean age of the participants was observed to be 15.26±3.09 years (Table 1) and the demographic data of the participants in the study is reflective of the demographic of the clients in the clinic from the clinic attendance register. Sickle Cell Anaemia related demographic characteristics as shown in Table 2 revealed that 44.2% of the adolescents were in one support group or the other. Fifty-eight (58) adolescents said they have other siblings living with SCA. Also results showed that more than half (62.5%) of the participants that participated in the study had SCA crisis in the last six months while 46.2% said they were admitted into the hospital within the last six months as a result of SCA related crises. The mean SCA knowledge of participants in the study as shown in Table 3 was observed to be 22.12±2.76 on the 100-percentile scale. Only 15.4% of the participants had good knowledge of SCA while more than half (57.7%) had poor knowledge. Half of the participants had moderate quality of life while only 13 (12.5%) have high quality of life. The mean score of the participants on the quality of life scale was observed to be 39.50±6.47 on the 100-percentile scale (Table 4). Findings showed that, been treated differently by teachers (3.29±0.97); been teased by other adolescents (3.22±0.92); troubled listening in class (2.93±1.02); felt worried (2.88±1.09); and pressured by friend (2.88±1.06) were issues that affected the adolescents quality of life most (Table 5).

**Table 1:** Respondents’ socio demographic characteristics

		Frequency (n = 104)	Percentage (%)
Gender	Male	66	63.5
	Female	38	36.5
Religion	Christianity	81	77.9
	Islam	23	22.1
Ethnicity	Yoruba	95	91.3
	Hausa	7	6.7
	Igbo	1	1.0
	Others	1	1.0
Age in years	10-14	37	35.6
	15-16	23	22.1
	17-19	44	42.3
	Mean	15.26 ± 3.09	

**Table 2:** Sickle cell disease related demographic characteristics

		Frequency (n = 104)	Percentage (%)
I am in a support group	Yes	46	44.2
	No	58	55.8
Number of siblings with SCD	Nil	46	44.2
	1-2	49	47.1
	3-5	9	8.7
	Mean	0.89 ± 1.04	
When last I had SCD crises in months	1-6	65	62.5
	7-12	28	26.9
	13-24	2	1.9
	25 and above	9	8.7
When last I was admitted into the hospital as a result of SCD in months	1-6	48	46.2
	7-12	30	28.8
	13-24	11	10.6
	25 and above	15	14.4
Number of SCD crises in a year on the average	1-2	62	59.6
	3-5	26	25.0
	6 and above	16	15.4
Number of SCD crises in the last 3 months	Nil	41	39.4
	1-2	50	48.1
	3-5	10	9.6
	6 and above	3	2.9

**Table 3:** Knowledge of respondents about sickle cell disease

	Frequency (n = 104)	Percentage (%)
Good knowledge	16	15.4
Fair knowledge	28	26.9
Poor knowledge	60	57.7
Mean (total 30)	22.12±2.76	

**Table 4:** Categorization of the quality of life of the respondents

	Frequency (n = 104)	Percentage (%)
High Quality of Life	13	12.5
Moderate Quality of Life	52	50.0
Low Quality of Life	39	37.5
Mean (total 60.00)	39.50±6.47	

**Table 5:** Mean score on the quality of life of the respondents

Question Items	Mean
I was treated differently by my teachers	3.29±0.97
I was teased by other kids	3.22±0.92
I had trouble listening in class	2.93±1.02
I felt worried	2.88±1.09
I felt pressure from my friends	2.88±1.06
I thought I was physically different from others of my age	2.87±1.03
I had low energy	2.80±0.99
I felt a dull, soft pain	2.78±1.00
I had difficulty controlling my pain	2.72±1.07
I could not keep up with my school work	2.64±1.09
I could not play when I wanted to	2.59±1.02
I took all my medication	2.09±1.14
I spent time with friend	2.06±0.93
I was able to engage in normal activities with friends	1.91±1.03
I felt good about myself	1.85±1.01

## Discussion

This study assessed quality of life of adolescents living with sickle cell disease in Ondo State, Southwest, Nigeria. The demographic characteristics of the respondents showed that most adolescents in this study were males, which supports findings of Amr, and colleagues [11] where they found out that male adolescents with SCA were more than female adolescents. This is also corroborated by submission of Fernande *et al.* in a study carried out among patients with SCA from northeastern region of Brazil [12]. However, findings of present study contradict findings of Jaffer *et al.* where more female participants were documented in a study that assessed knowledge and preventive measure towards SCA crises among Bahraini adult living with sickle cell disease [13]. The mean age of the participants in this study was observed to be 15.26±3.09 years; this age group was among the modal age suggested by Fernande *et al.* (2015) among participants in their study [12]. Findings on participants' knowledge about SCA revealed that majority of the adolescent had poor knowledge (57.7%) of the disease. This is consistent with submissions of previous researchers where they reported low level of knowledge of SCA transition among people living with SCA [14]. Although it is expected that the level of knowledge in the present study should be better than that of Acharya, Walsh, and Friedman (2009) study. This is because while Acharya, Walsh, and Friedman conducted their study among the general population including those without sickle cell anaemia, the present study focused only on those with SCA [14]. This study however, observed that having SCA does not necessarily make people to have better knowledge compared to those without SCA. However, the differences in the settings where our study and previous studies were carried out might have also accounted for this.

In an earlier study carried out among local government workers in Ile-Ife, Nigeria Abioye-Kuteyi *et al*. submitted that 69% of the workers that participated in their study have poor knowledge of sickle cell disease [15]. However, in a study carried out among people with SCD, Jaffer and colleagues submitted that participants in their study were moderately knowledgeable about SCD [13]. Half of the participants in this study were found to have moderate quality of life that is scoring between 25^th^ to 50^th^ percentiles of the quality of life scale. This is lower than what was reported among adult with sickle cell disease by Mann-Jiles V *et al*. (2010) among patients with SCA in a General Hospital of Goiás, Brazil [16]. Being teased by friends; troubled listening in class; felt worried; and pressured by friend were observed to be things that affected the adolescents quality of life negatively the most. This can mean that good network support especially by friends might play an important role in improving the quality of life of adolescent living with sickle cell diseases. This is closely associated with the submission Acharya, Walsh, and Friedman that social stigma is one of the issues affecting people living with SCD [14]. Roberti *et al*. also submitted that sickle cell disease is usually seen as stigma [17]. Our results had also been previously corroborated by separate submission of, Jenerette and Brewer; and Desai and Serjeant [18,19]. In this study, been teased by friends was found to be the most frequent issue raised by the participants affecting their quality of life. This is similar to feelings by the people as reported among patients with sickle cell disease. In a study by Acharya, Walsh and Friedman Ross (2009), they found out that some participants in their study said they are careful about who they reveal their SCA status to, because of fear of stigma [14]. Amr, Amin and Al-Omair in an attempt to evaluate health related quality of life among adolescents with sickle cell disease in Saudi Arabia found out that there is a significant relationship between gender and the adolescent quality of life [11]. Their study found out that female gender had worse quality of life compared to their male counterpart. The present study established a weak negative correlation between gender and quality of life of adolescents that participated in the study. This correlation, however, was observed not to be significant.

## Conclusion

Sickle cell disease had been documented to reduce the quality of life of people living with it. This study established that knowledge of adolescents with SCD about SCD is low and their quality of life is moderate. Hence, there is need to improve the knowledge of people living with SCD which will ultimately improve their quality of life.

### What is known about this topic

- SCA affects many people in the developing nations of the world like Nigeria;
- SCA places high burden on family and caregivers.

### What this study adds

- Quantitative assessment of quality of life of adolescents living with SCA;
- Level of knowledge of adolescents living with SCA about SCA;
- Relationship between level of knowledge of adolescent about SCA and their quality of life.

## Competing interests

The authors declare no competing interests.
